# Protocol for intervention-free quantification of protein turnover rate by steady-state modeling

**DOI:** 10.1016/j.xpro.2021.100377

**Published:** 2021-03-18

**Authors:** Stefania Marcotti, Besaiz Jose Sánchez-Sánchez, Eduardo Serna-Morales, Anca Dragu, María-del-Carmen Díaz-de-la-Loza, Yutaka Matsubayashi, Brian Marc Stramer

**Affiliations:** 1 Randall Centre for Cell and Molecular Biophysics, King’s College London, London SE1 1UL, UK

**Keywords:** Bioinformatics, Cell biology, Model organisms, Molecular biology, Protein biochemistry

## Abstract

Protein turnover rate is difficult to obtain experimentally. This protocol shows how to mathematically model turnover rates in an intervention-free manner given the ability to quantify mRNA and protein expression from initiation to homeostasis. This approach can be used to calculate production and degradation rates and to infer protein half-life. This model was successfully employed to quantify turnover during *Drosophila* embryogenesis, and we hypothesize that it will be applicable to diverse *in vivo* or *in vitro* systems.

For complete details on the use and execution of this protocol, please refer to Matsubayashi et al. (2020).

## Highlights

• Protocol for intervention-free modeling of protein turnover rates
• Based on quantifying mRNA and protein levels from initiation to homeostasis
• Devised for inferring protein half-life in fly embryos but applicable across systems

## Graphical abstract

**Figure:**
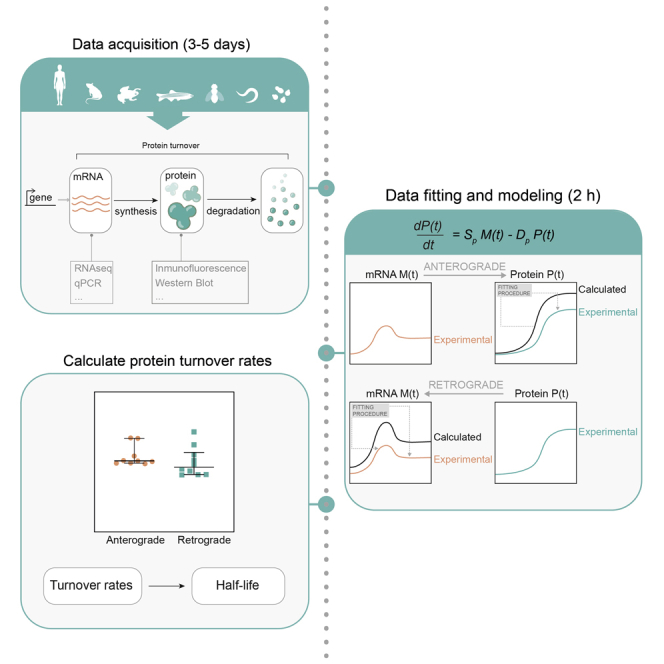

## Before you begin

### What data do you need?

This protocol requires the temporal expression of a protein of interest and its mRNA profile as input data to quantify turnover. Both should be observed over the same time frame, starting as close as possible to initiation of expression, until as close as possible to reaching homeostasis. The protein and mRNA profiles do not need the same temporal resolution (i.e., they can have different sampling intervals) and can be obtained using a number of different experimental approaches. Two datasets are used as examples in this protocol, related to Collagen IV (ColIV) and Nidogen (Ndg) expression in the *Drosophila* embryo (see Material S3). mRNA levels were quantified from an RNA-seq time course (Graveley et al., 2011) and protein levels were obtained by measuring the fluorescence intensity of GFP protein-trap lines during development (Matsubayashi et al., 2020) (see the data acquisition section for more details). It should be noted that this approach is not limited to *in vivo* models and to the experimental methods used here. For example, a similar approach could be used to analyze protein turnover in cultured cells or isolated tissues, provided that there are methods to experimentally obtain information on the temporal dynamics of the protein of interest and its mRNA profile.

### Modeling hypotheses

The model presented in this protocol relies on the assumption that the protein expression over time is controlled by a single rate of synthesis and a single rate of degradation, both constant over time, which is a common hypothesis of most experimental analyses of protein turnover (Beynon, 2005; Claydon and Beynon, 2012; Hinkson and Elias, 2011; Kristensen et al., 2013; Schwanhäusser et al., 2013; Tchourine et al., 2014) (refer to the limitations section for further details).

The model can be mathematically described as follows:

Equation 1 $$\frac{dP\left(t\right)}{dt}=S_{p}M\left(t\right)-\;D_{p}P\left(t\right)$$

where P(t) is the protein expression over time t, M(t) is the mRNA profile over time, S_p_ is the constant rate of protein synthesis, and D_p_ the constant rate of protein degradation (Matsubayashi et al., 2020; Tchourine et al., 2014). This corresponds to hypothesizing that the net change in protein levels over time is determined by synthesis minus degradation, with the amount of synthesis and degradation proportional to the mRNA levels and the protein levels, respectively. The aim of this protocol is to calculate the synthesis and degradation rates, using as input experimental data for the expression levels of RNA and protein. From the degradation rate, the protein half-life can be subsequently inferred as the ln(2) divided by the degradation rate itself (Equation 5) (Claydon and Beynon, 2012; Matsubayashi et al., 2020).

**Figure 1:**
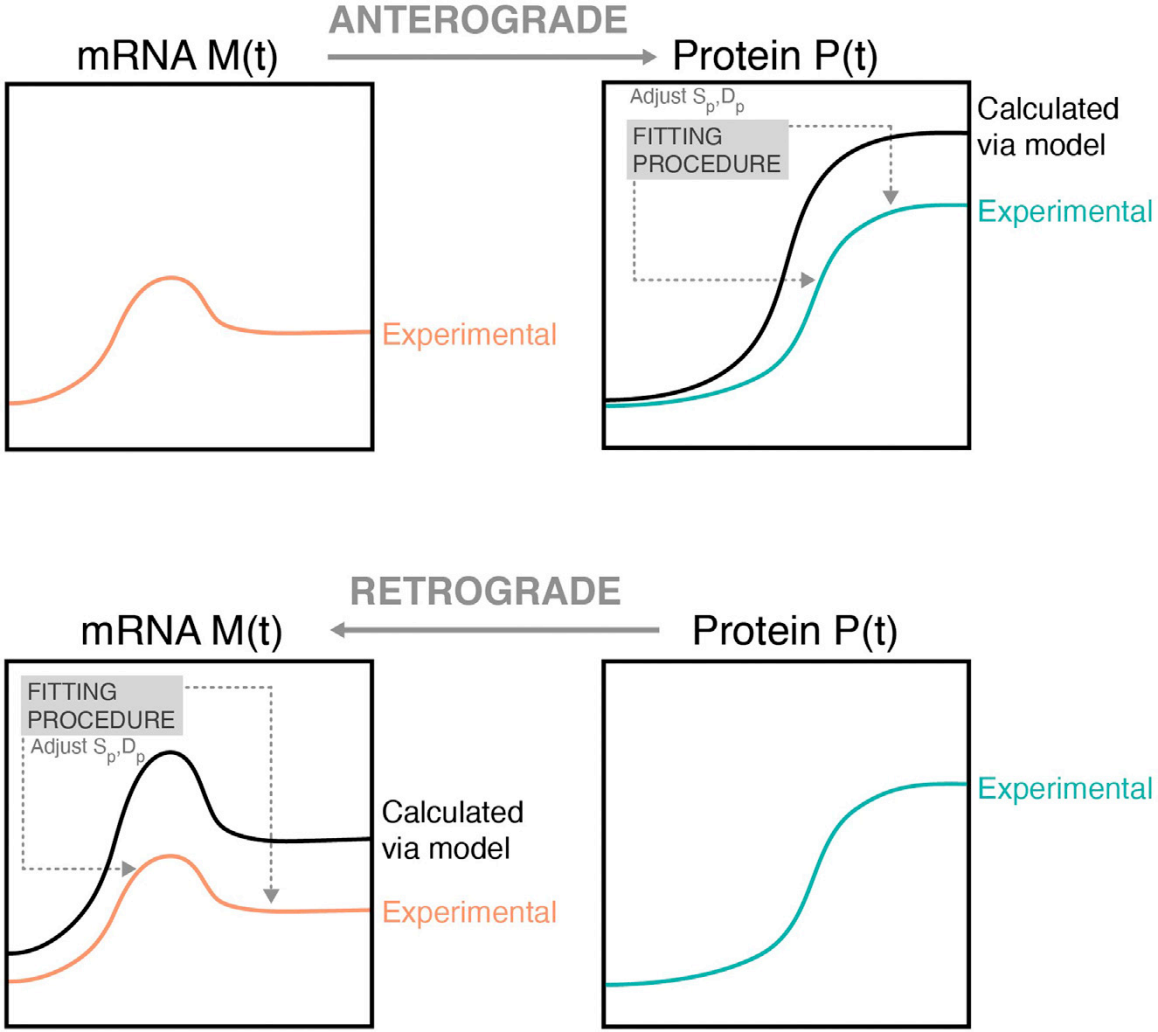
Anterograde and retrograde modeling Schematic of the anterograde (mRNA to protein) and retrograde (protein to mRNA) modeling. For the anterograde (retrograde) model, the mRNA (protein) measured experimentally is used as input to calculate the protein (mRNA) dynamics. The obtained protein (mRNA) profile undergoes a fitting procedure to find the best parameters for the synthesis and degradation rates to match the experimentally observed protein (mRNA) dynamics.

Equation 1 can be solved either numerically for the protein levels by using as input the experimental mRNA data or analytically for the mRNA levels by using as input the experimental protein expression. We named the first approach “Anterograde modeling” (i.e., from mRNA to protein expression), and the second approach “retrograde modeling” (i.e., from protein expression to mRNA). In both cases, the synthesis and degradation rates can be found by nonlinear regression between the calculated solution and the corresponding experimental data (Figure 1, see the anterograde modeling and retrograde modeling sections for more details).

### Data acquisition

**Timing: 3–5 days**

**Figure 2:**
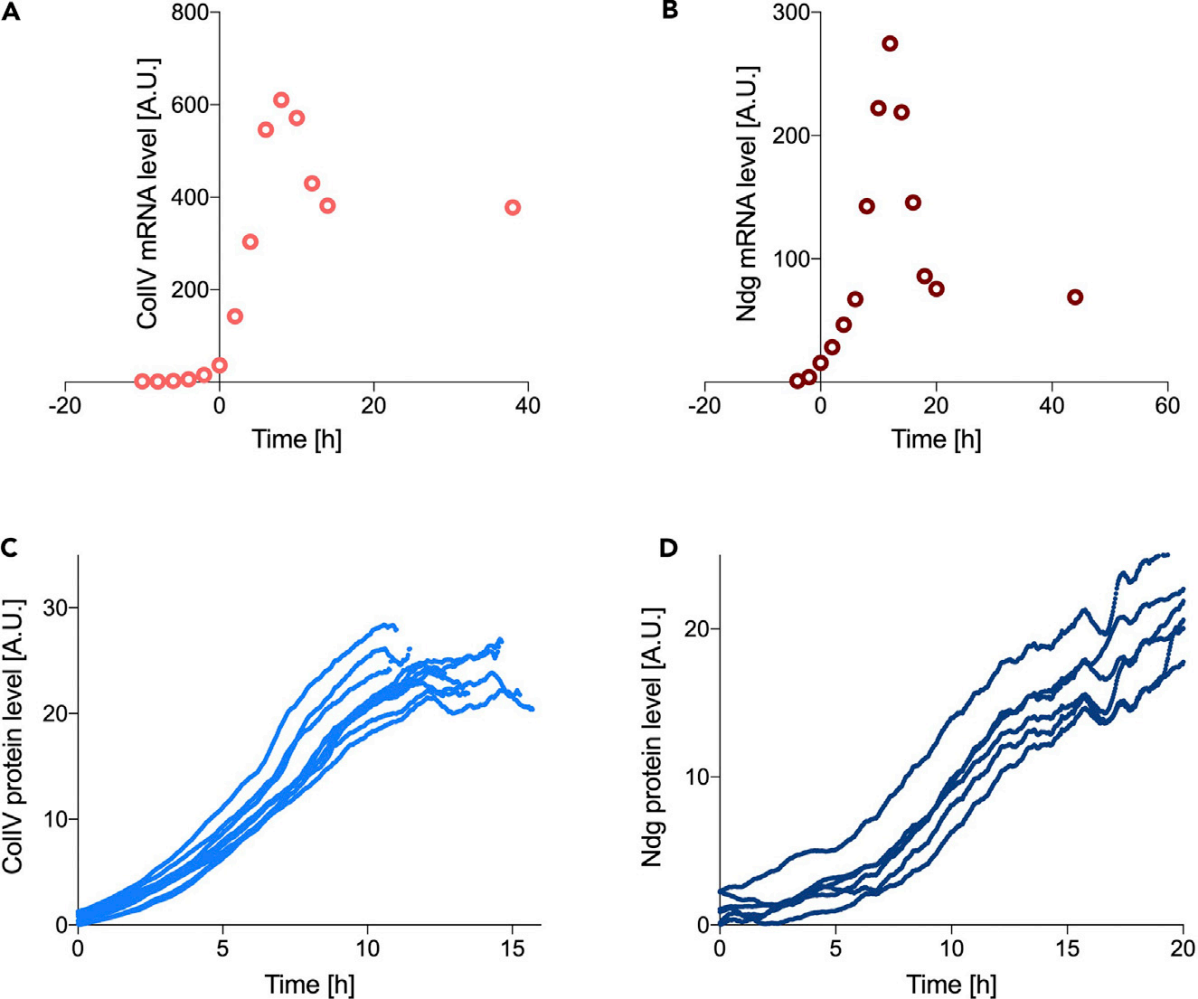
Experimental input data for modeling Smoothed mRNA temporal profile for ColIV (A) and Ndg (B), respectively, obtained by an RNA-seq time course of *Drosophila* development. ColIV (C) and Ndg (D) protein expression acquired experimentally by time-lapse images of viable GFP protein-trap lines in the *Drosophila* genes ColIV and Ndg. Each curve represents a biological replicate (n = 10 ColIV, n = 6 Ndg). A.U., arbitrary units (relative measure over time). Data are available in the Material S3.

***Note:*** Both the ColIV and Ndg profiles were smoothed with a walking average of 3 data points. Smoothing should be considered on a case-by-case basis and kept consistent across multiple samples within an experiment. Smoothing is necessary when poor fitting is obtained at step 2 of Data fitting (see also troubleshootingproblem 1) to help with cases when the time course is sparse and does not give a complete picture of the curve shape and to reduce the noise from the experimental data.

***Note:*** Please refer to troubleshootingproblem 2 should more than one mRNA time course be available.

1. Measure the mRNA time course for the protein of interest. This can be obtained by many different methods, (e.g., RNA-seq or qPCR), to show relative changes in mRNA expression over time. For the purpose of this protocol, we used the mRNA temporal profile for ColIV and Ndg, which were obtained through an RNA-seq time course of *Drosophila* development (http://flybase.org, Figures 2A and 2B, see Material S3) (Graveley et al., 2011; Matsubayashi et al., 2020); similar databases are available for other species, such as Wormbase for *C. elegans* and related nematodes (http://wormbase.org/).

2. Measure the protein expression time course. The relative changes in the temporal expression of a protein of interest can be acquired, for example, by taking time-lapse images of a fluorescently labeled protein in a model organism or via western blot experiments. The choice of the most suitable experimental method to acquire such measurements ultimately depends on the model system of interest. Western blots, despite offering a direct measurement of the protein level, are experimentally more challenging and time consuming as they require multiple harvesting at different time points. The achievable temporal resolution might therefore be worse than imaging. Conversely, if it is not possible to fluorescently tag the protein or to observe it in the tissue of interest, or where concerns with imaging acquisition or fluorophore maturation lags arise, western blots should be preferred.

***Note:*** For the purpose of this protocol, we used time-lapse images of viable GFP protein-trap lines in the *Drosophila* ColIV and Ndg (Morin et al., 2001), as we were interested in looking at ECM turnover (Matsubayashi et al., 2020). It should be noted that embryos are not required to be homozygous for the protein-trap, provided that the fluorescence intensity is strong enough for imaging quantification. Moreover, the protein should be endogenous or driven by its own promoter and it should be verified that the associated fluorophore is stable and matures quickly enough, as the protein amount is indirectly measured via the proxy of fluorescence intensity. The maturation time of GFP is relatively fast (∼14–60 min) (Iizuka et al., 2011) compared to the observed dynamics in our proteins of interest and was therefore deemed suitable; however, fast-folding GFP could be considered for faster processes and dynamics.

***Note:*** Whole embryos were imaged every 2 min under a dissection microscope to observe the increase in fluorescence from induction to homeostasis (Figures 2C and 2D, see Material S3) (Matsubayashi et al., 2020). This imaging modality was chosen due to the ease of gathering a large amount of data simultaneously, however, confocal or widefield microscopy would be equally valid. Relative changes in fluorescence intensity over time should be acquired (arbitrary units). Fluorescence quantification for the example dataset (Matsubayashi et al., 2020) was performed as follows. Fluorescent protein-expressing embryos were imaged together with control embryos not expressing the fluorophore. The average raw fluorescence intensity in each embryo at each time point was measured in Fiji and the acquired curve smoothed with a 15-frame moving average. To remove the contribution of embryonic autofluorescence, the signal from control embryos was subtracted from the experimental sample at each timepoint.

**CRITICAL:** if using fluorescently labeled proteins, the effects of photobleaching should be determined. This can be done by comparing the fluorescence of two samples, one of which is imaged following the experimental protocol (e.g., once every 2 min) and the other is spared. The effect of photobleaching could be considered negligible if the two samples displayed similar fluorescence levels at the end of the experiment. Refer to the troubleshootingproblem 3 section for more information.

### Modeling requirements

**CRITICAL:** Check the mRNA profile. The model assumes a simple relationship between mRNA and protein expression (i.e., a single constant rate of synthesis). For this reason, a first warning sign that the model might not be appropriate for a specific case is if the mRNA temporal expression displays a complex profile (e.g., multiple peaks, which suggest the presence of multiple rates of synthesis or of time-dependent rates, see limitations section, Figure 8). The sample mRNA profiles for *Drosophila* ColIV and Ndg display a simple behavior, with a single peak followed by a plateau (Figures 2A and 2B), and they are therefore suitable for this modeling approach. The presence of a plateau is expected when the process reaches homeostasis, i.e., an equilibrium state.

**CRITICAL:** Check the protein expression profile. The protein expression is expected to follow a logistic behavior over time, hence showing a sigmoid or S-shape starting from zero and ending with a plateau. If the protein of interest does not follow such a trend, this is a second warning sign that the model described in this protocol might not be sufficient to calculate turnover rates (see limitations section). This requirement is met in the sample datasets, as the temporal expression of GFP-tagged ColIVa2 and Ndg follow a logistic trend (Figures 2C and 2D).

### Code and folders setup

3. Download the code from https://github.com/stemarcotti/protein_turnover_modelling, by clicking on the green button “Code” and selecting “Download ZIP” to save the file locally on your machine. Unzip the folder.
4. Open MATLAB and navigate to the unzipped folder where the code is located. Further assistance on how to do so can be found here. The code was tested on MATLAB v.2018b.

## Key resources table

**Table:** 

REAGENT or RESOURCE	SOURCE	IDENTIFIER
**Experimental models: organisms/strains**		

*D. melanogaster:* ColIVα2 (Vkg)-GFP (embryos)	(Morin et al., 2001)	N/A
*D. melanogaster: Ndg-GFP* (embryos)	(Sarov et al., 2016)	N/A

**Software and algorithms**		

LAS AF	Leica	http://www.leica-microsystems.com/home/
ImageJ/Fiji	Fiji	http://fiji.sc/
MATLAB R2018b	MathWorks	https://www.mathworks.com/products/matlab.html
Prism (8 or 9)	GraphPad	https://www.graphpad.com/scientific-software/prism/
MATLAB custom code for fitting purpose	(Matsubayashi et al., 2020)	https://github.com/stemarcotti/protein_turnover_modelling

**Deposited data**		

The “modENCODE Temporal Expression Data” of *vkg/ColIVα2* mRNA	(Graveley et al., 2011)	http://flybase.org/reports/FBgn0016075
The “modENCODE Temporal Expression Data” of *LanA* mRNA	(Graveley et al., 2011)	http://flybase.org/reports/FBgn0002526
The “modENCODE Temporal Expression Data” of *Ndg* mRNA	(Graveley et al., 2011)	https://flybase.org/reports/FBgn0026403

**Other**		

M205 fluorescent dissection microscope	Leica	http://www.leica-microsystems.com/home/
PLANAPO 2.0× objective for M205	Leica	10450030

***Alternatives:*** Live organism, tissue, cultured cells, etc. for which it is possible to measure mRNA and protein profile over time can be used as the experimental models.

***Alternatives:*** Any Imaging software other than LAS AF can be used to quantify fluorescent proteins.

***Alternatives:*** Any statistical software for fitting and graphing (e.g., SPSS, Prism, R, MATLAB, Python) can be applied for data analysis.

***Alternatives:*** Depending on the model system, another experimental setup may be required to measure the mRNA and protein profiles over time (e.g., microscope and objectives).

## Step-by-step method details

### Data fitting

**Timing: 1 h**

1. Temporal resolution considerations. Due to experimental constraints, it is possible that the temporal resolution of the mRNA and protein measurements may not be identical (e.g., the mRNA sampling is performed less often than the fluorescence time-lapse imaging). If this is the case, the mRNA profile should be interpolated to obtain information on the intermediate time points. The mRNA profiles for *Drosophila* ColIV and Ndg was sampled with 2 h intervals starting from egg laying, and it was interpolated to obtain a data point every 2 min to match the sampling frequency of the protein. There is no theoretical limit on how different the temporal resolutions could be; however, the sampling frequency should be enough to give confidence that the interpolated curve is reflecting the observed dynamics (e.g., if two time points are too far from each other, the curve in between could take a number of shapes).

**CRITICAL:** care should be taken to align the temporal scales of both mRNA and protein data. The imaging for ColIV started at embryonic stage 15 (about 11 h 20 min after egg laying); therefore, the time was set to zero at this point for the ColIV mRNA data. The imaging for Ndg started at embryonic stage 11 (about 5 h after egg laying); therefore, the time was set to zero at this point for the Ndg mRNA data. Time zero on the two graphs in Figures 2A and 2B therefore represents two different stages of development.

**Figure 3:**
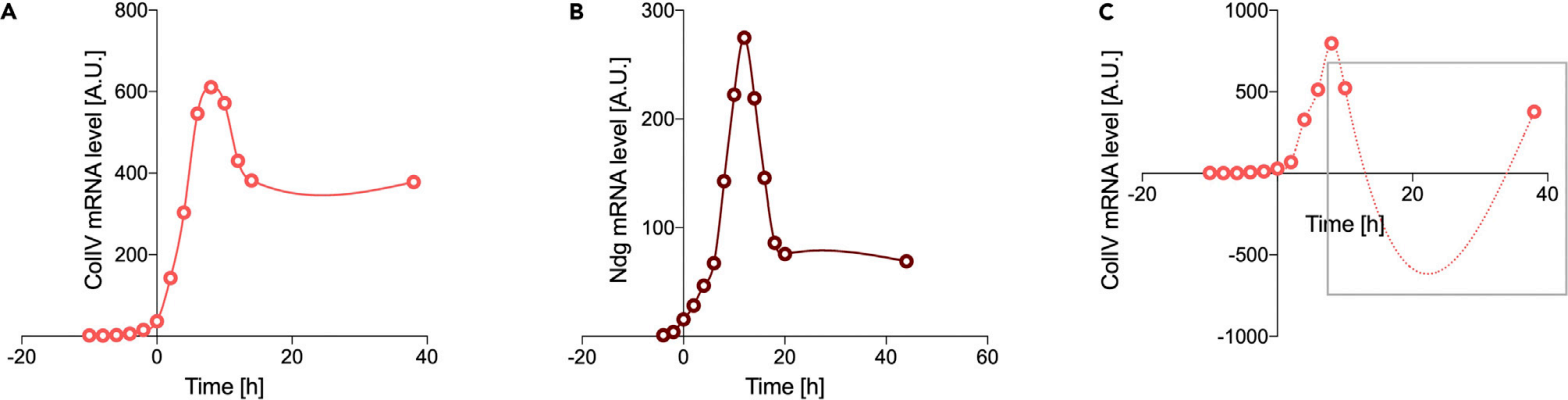
Fitting of mRNA temporal profile Spline interpolation (solid line) for ColIV (A) and Ndg (B) mRNA data, respectively, allowing to infer the intermediate data points between mRNA experimental acquisitions (circles). A poor fitting example is shown in (C): the data point at 12 h and 14 h were deleted to simulate poor fitting quality for the unsmoothed ColIV mRNA profile with the same interpolating spline function as of (A) (dash line, gray square). The data points at later time are not frequent enough to capture the curve plateau at equilibrium. A.U., arbitrary units (relative measure over time).

2. mRNA profile interpolation. Different software can be used to interpolate the mRNA data to increase temporal resolution; an option is detailed below using GraphPad Prism (version 8 or 9). A standard spline fitting can be chosen when the raw data have relatively even spacing between time intervals. A spline provides piece-wise polynomial fitting of a curve by dividing it in a number of segments. An increase in the number of segments and an increase in the temporal resolution of the data will increase the spline fitting quality.
  a. Copy time and mRNA profile data into an XY data table in Prism
  b. Calculate how many data points are needed in the interpolated function. In this case we want to match the experimental protein acquisition to obtain a data point every 2 min. The total available time we have ColIV and Ndg mRNA data for is −10 h to 38 h and −-4 h to 44 h, respectively (48 h = 2,880 min; time zero is the start of protein expression measurements). Therefore, we need the interpolated function on 1,441 data points (2,880/2 min + 1 data point for t = 0).
  c. Select “Analyze > Fit spline/LOWESS” from the Analysis menu
  d. A cubic spline with 1,441 segments was chosen for this example (Figures 3A and 3B), but the parameters might need to be adjusted for different data to assure good fitting quality (Figure 3C).

**CRITICAL:** check your results for overall fitting quality (see also troubleshootingproblem 1). An example of poor fitting quality for the ColIV mRNA profile is shown in Figure 3C, where the fitting does not match the expected curve shape with a peak and a plateau. The available data are too sparse at late time points to fully capture the mRNA dynamics at equilibrium (i.e., plateau). It is worth noting that acquiring the ColIV example data beyond 15 h is essential simply to make sure that the mRNA profile is reaching homeostasis around the time when we observe the protein levels also reaching equilibrium.

**Figure 4:**
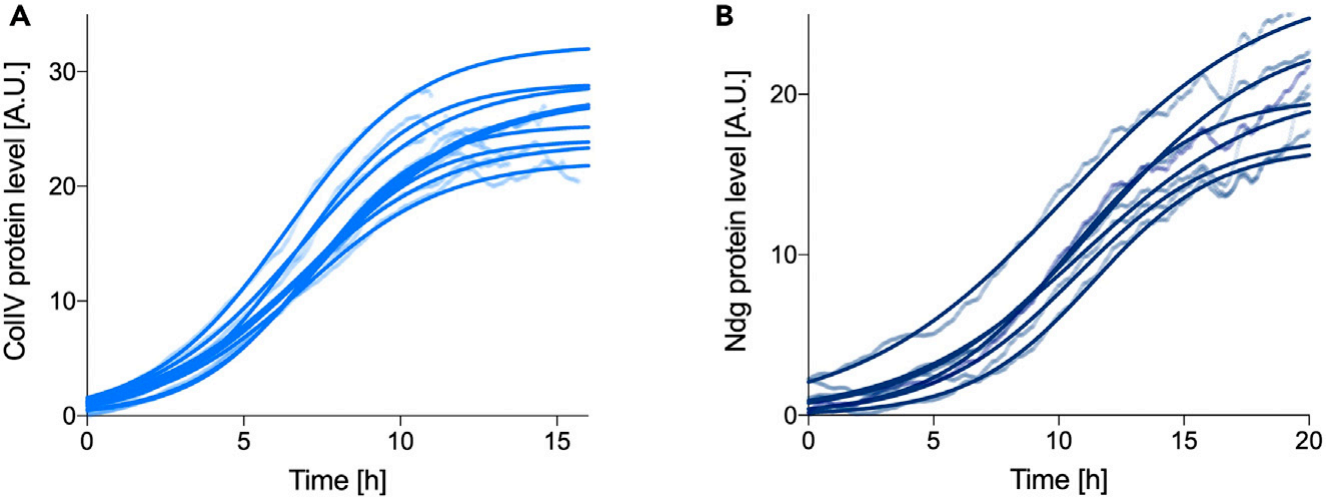
Fitting of protein temporal profile Logistic fitting (solid line) of ColIV (A) and Ndg (B) protein levels (semi-transparent dots) for all available biological replicates (n = 10 ColIV, n = 6 Ndg). A.U., arbitrary units (relative measure over time).
  e. The output parameters are linked to the logistic parameters K, t_i_ and r as follows:

3. Protein expression fitting. Protein expression is expected to show a logistic trend over time from initiation to homeostasis for the purpose of the present modeling (refer to step 4 of Modeling requirements for details). Logistic curves can be fully described by three parameters (Brown and Rothery, 1993): the carrying capacity K (value at infinite time), the inflection point t_i_ (midpoint of the curve), and the intrinsic rate of increase r (steepness of the curve around t_i_). Logistic fitting can be achieved in GraphPad Prism as follows.
  a. Copy time and protein expression data into an XY data table, with one column for each experimental replicate
  b. Select “Analyze > Nonlinear regression (curve fit)” from the Analysis menu
  c. Select the option “log(agonist)vs. response -- Variable slope (four parameters)” in the Dose-response – Stimulation sub-menu
  d. In the Results page, check the Goodness of Fit – R squared row, to evaluate how well the data fit to a logistic curve. Values close to one signify best fitting (an average R squared of 0.99 was achieved with both sample datasets, Figures 4A and 4B). It is not possible to state a cut-off value on the R squared parameter, and fitting quality should always be visually evaluated against the expected dynamics (i.e., curve shape).

Equation 2 K=Span

Equation 3 $$t_{i}=\log{\text{EC}}_{50}$$

Equation 4 r=HillSlope∗ln(10)

A fourth parameter called Bottom is provided; this represents the starting value of the logistic curve and it is expected to be close to zero. If protein levels are obtained by imaging of a fluorescently tagged protein, the Bottom parameter represents the residual autofluorescence. To facilitate the conversion between the GraphPad parameters and the logistic parameters an Excel workbook named 01_logistic_parameters.xlsx is provided in the Material S1.

**CRITICAL:** fitting quality scores, such as R^2^, should be checked. If the performed fitting has low quality, it is likely that the protein does not follow a logistic behavior, or that not enough data points were obtained within the time interval of interest to fully capture the logistic trend. This could mean that the data acquisition should be adjusted (see what data do you need? and data acquisition) or that the protein of interest does not meet the modeling assumptions (see modeling hypotheses and limitations sections).

### Anterograde modeling

**Timing: 30 min**

**Figure 5:**
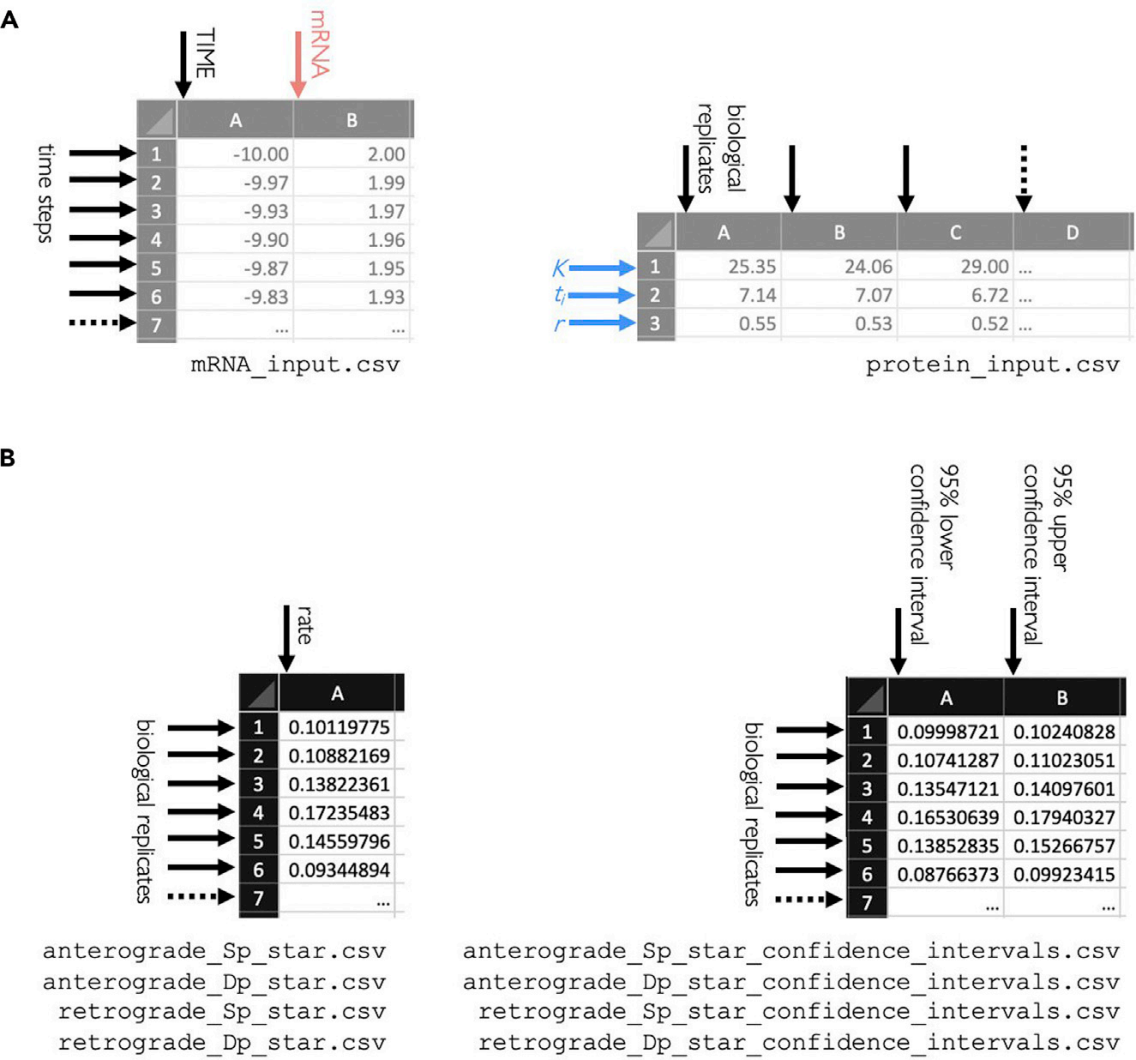
Structure of input and output files Schematic of the required input (A) and the obtained output files (B).
  c. In MATLAB, open the file called anterograde_model.m and hit Run. The code will request you to locate the two files you created and to choose the time interval and span of your experiments (in the case of the sample data, Time interval [min] = 2 and “Starting time experiment [h]” and “Finishing time experiment [h]” were chosen equal to 0 and 15 for ColIV and to 0 and 20 for Ndg, respectively).
  d. The code will produce four output csv files, containing the values for the synthesis and degradation rates and their confidence intervals (Figure 5B). Each row represents a biological replicate.

4. Run anterograde modeling. The model in Equation 1 can be solved numerically for the protein expression if using the mRNA temporal profile as input (Matsubayashi et al., 2020). The synthesis and degradation rates can be obtained by minimizing the difference between the numerical solution and the experimentally measured protein levels. This is achieved by nonlinear regression (Levenberg-Marquardt nonlinear least squares algorithm, MATLAB function nlinfit); confidence intervals for the fitted parameters are also computed.
  a. Create an empty folder where the analysis output will be saved
  b. Inside the folder, create two separate csv files with the interpolated mRNA profile (step 2 of data fitting) and the fitted logistic parameters for the protein profile (step 3 of data fitting). The first file should include two columns, one for time and one for mRNA values, and the number of rows will depend on the chosen time steps and interval; the second file should include three rows (K, t_i_, r) and one column for each biological replicate (Figure 5A). The number of biological replicates to include ultimately depends on the biological and experimental variability in the system used. In this example, due to small discrepancies in embryo staging and possible fluctuations in room temperature (24°C–25°C), 10 ColIV and 6 Ndg samples were deemed sufficient.

**CRITICAL:** the confidence intervals should be checked in order to verify good fitting of the model. These values are expected to be much smaller than the biological variability between replicates (see also troubleshootingproblem 4).

### Retrograde modeling

**Timing: 30 min**

5. Run retrograde modeling. The model in Equation 1 can be solved analytically for the mRNA profile if using the logistic curve fitted to the experimentally measured protein expression as input (Matsubayashi et al., 2020). The synthesis and degradation rates can be obtained by minimizing the difference between the analytical solution and the experimentally obtained mRNA profiles. This is achieved with the same nonlinear regression algorithm of the anterograde case; confidence intervals for the fitted parameters are computed.
  a. Create an empty folder where the analysis output will be saved
  b. Copy in the newly created folder the two csv files created at step 4b of Anterograde modeling
  c. In MATLAB, navigate to the folder where the code was saved. Open the file called retrograde_model.m and hit Run. The code will request you to locate the two files you copied and to choose the time interval and span of your experiments (in the case of the sample data, Time interval [min] = 2 and “Starting time experiment [h]” and “Finishing time experiment [h]” were chosen equal to 0 and 15 for ColIV and to 0 and 20 for Ndg, respectively).
  d. The code will produce four output csv files, containing the values for the synthesis and degradation rates and their confidence intervals (Figure 5B). Each row represents a biological replicate.

**Figure 6:**
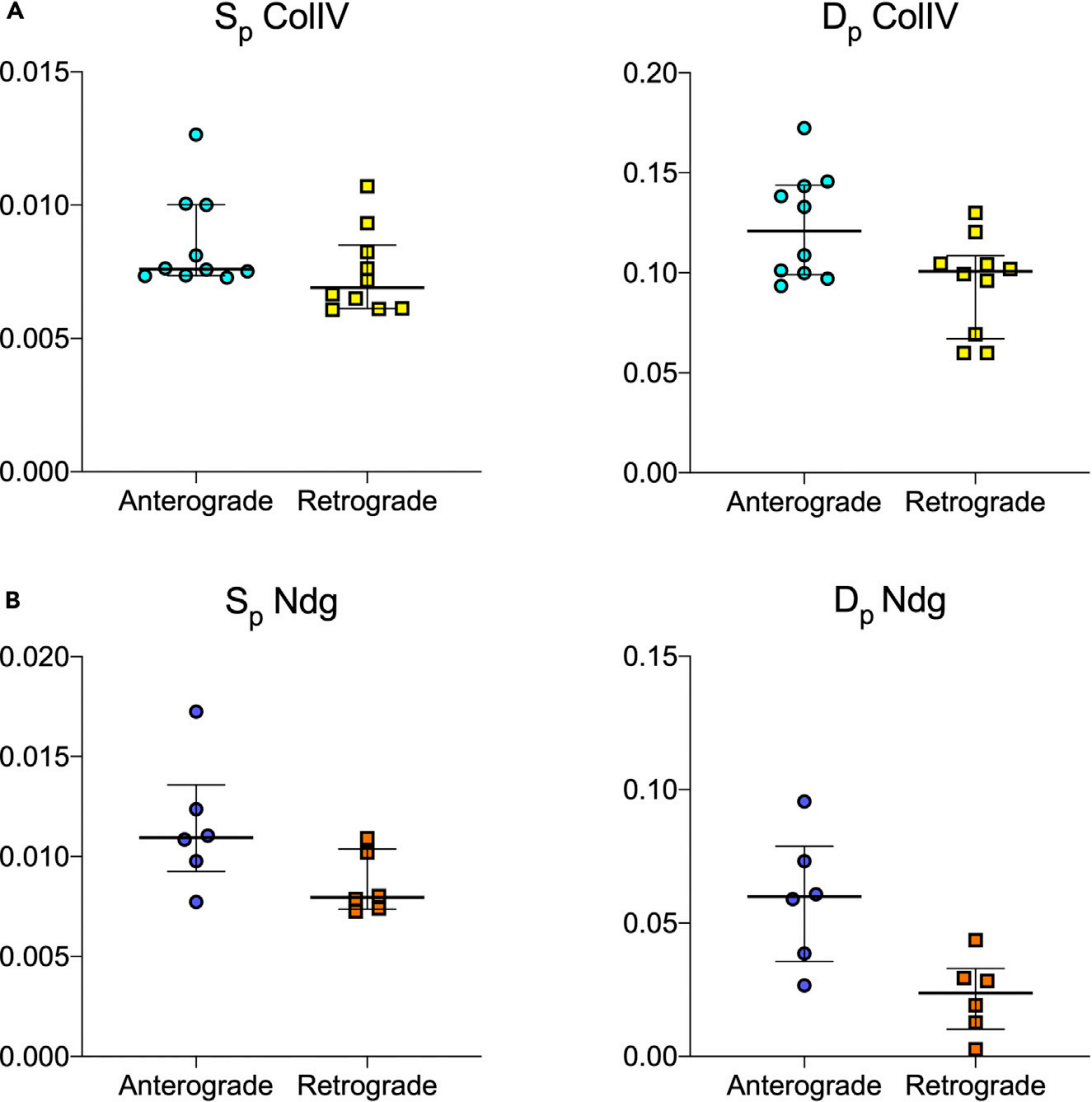
Modeled turnover rates Comparison of ColIV (A) and Ndg (B) turnover rates obtained by anterograde and retrograde modeling for synthesis (S_p_) and degradation (D_p_). Each dot represents modeling for a biological replicate, bars represent median and interquartile range of the sample.

**CRITICAL:** this step represents a control for the internal consistency of the modeling, as the synthesis and degradation rates for anterograde and retrograde should be similar. If this is not the case, this is the third warning sign that the modeling might not be sufficient for the protein of interest (see the limitations section). In the case of the test data, this condition is satisfied, as the calculated turnover rates do not show statistical difference when comparing anterograde and retrograde modeling (Mann-Whitney two-tailed test, level of significance set to 0.01, for both ColIV and Ndg, Figures 6A and 6B).

**CRITICAL:** the confidence intervals should be checked in order to verify good fitting of the model. These values are expected to be much smaller than the biological variability between replicates (see also troubleshootingproblem 4).

### Calculate half-life

6. Protein half-life *h* is calculated as follows

Equation 5 $$h=\;\frac{\ln\left(2\right)}{D_{p}}$$

To facilitate the protein half-life calculation, an Excel workbook is provided as the Material S2, named 02_half-life.xlsx. To use, copy the degradation rate values obtained with anterograde modeling. Please note that this is an arbitrary choice, the degradation rates from the retrograde model could be used and should lead to similar results as internal consistency between the two modeling approaches is expected. The test data show an average protein half-life of about 6 h for ColIV and about 14 h for Ndg.

## Expected outcomes

**Figure 7:**
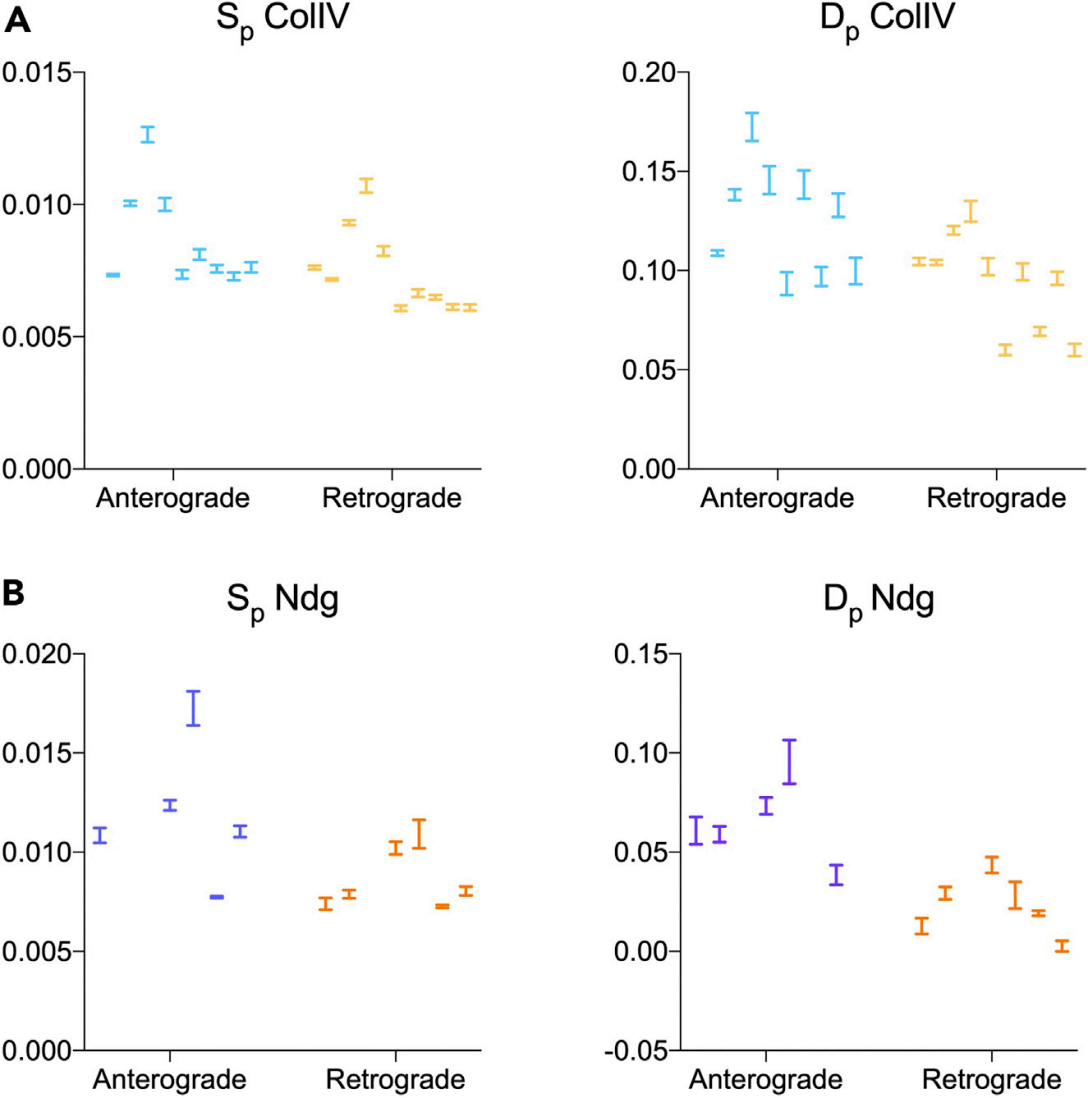
Confidence intervals of the modeled turnover rates Confidence intervals of turnover rates (synthesis S_p_ and degradation D_p_) obtained by anterograde and retrograde modeling for ColIV (A) and Ndg (B). The bars represent the range between the 95% lower and upper confidence intervals for the mean values of each biological replicate shown in Figure 6.

If the modeling was appropriate for the protein of interest, the anterograde and retrograde approaches should lead to similar values for the rate of synthesis and degradation (Figure 6). Moreover, the confidence intervals for these parameters should be small compared to biological variability (i.e., the nonlinear regression performed well on the data, Figure 7). If this is the case, the calculated rates can be tested experimentally to verify model predictions (Hinkson and Elias, 2011). To this aim, different approaches can be taken. For example, *in vivo* pulse-chase experiments and fluorescent decay after photoconversion analysis were successfully compared with the model output for the ColIV dataset (Matsubayashi et al., 2020).

Once turnover rates are confirmed experimentally, the modelling approach presented in this protocol makes it easier to elucidate regulators of the turnover process. The relative changes in protein turnover rates can therefore be easily examined in an intervention-free manner using a variety of perturbations (e.g., mutants or RNAi), which would be extremely difficult using an experimental approach such as pulse-chase analysis (Matsubayashi et al., 2020).

## Limitations

The presented model relies on some assumptions that might not hold for all proteins of interest. This modeling might not be appropriate for noisy measurements, complex expression profiles or in the presence of additional translation and degradation regulators that make S_p_ and D_p_ change with time. Despite these caveats, it was shown to offer good turnover predictions for about one-third of all the proteins in yeast cells (Tchourine et al., 2014).

Care should be taken due to the possible unreliability of the results when:

1. the mRNA profile displays complex trends. Here an example for *Drosophila* Laminin A (LanA) is shown (Figure 8), where multiple peaks can be observed. Please note that it would not be acceptable to perform the modeling using only data from a shorter timespan (e.g., 5–20 h for LanA), as the mRNA profile has to be observed reaching homeostasis. Moreover, the modeling relies on the assumption of single constant rate of synthesis and degradation, which is likely not met in this case.

**Figure 8:**
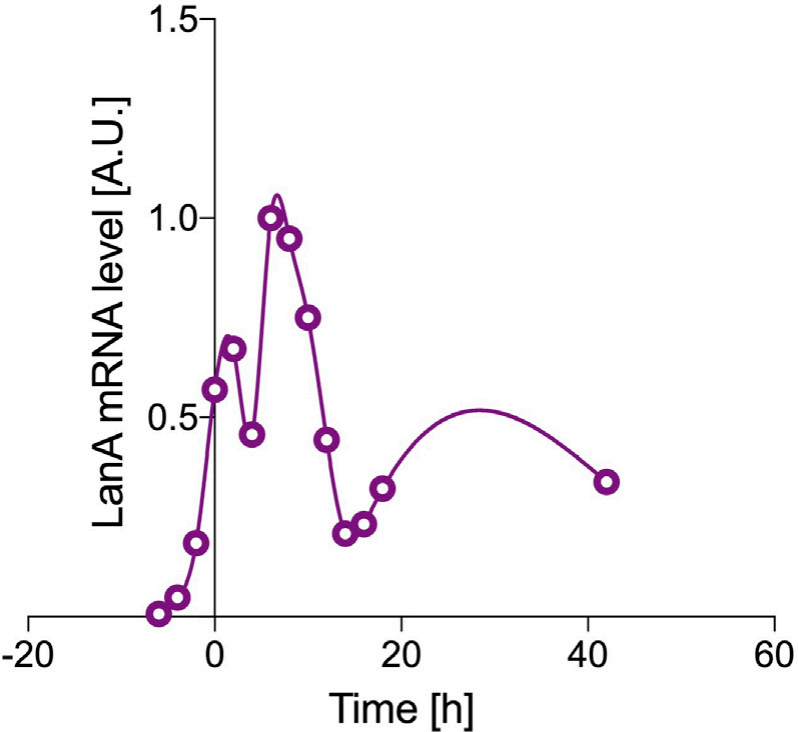
Example of unsuitable mRNA temporal profile mRNA levels for *Drosophila* LanA (circles) fitted with a cubic spline (solid line). It can be noted that the mRNA profile is complex, showing multiple peaks and failing to display a clear plateau at equilibrium. A.U., arbitrary units (relative measure over time).
2. the protein expression does not follow a logistic trend
3. the anterograde and retrograde model results do not show internal consistency

For successful modeling with the present protocol, protein expression should be quantified as close as possible from initiation to homeostasis. It is theoretically possible to take an opposite approach relying on similar assumptions to the ones presented here and quantify protein levels after inhibiting protein synthesis, observing expression during decay from homeostasis instead of during the increase to homeostasis, such as in metabolic radioisotope labeling experiments (Beavan et al., 1989; Cohen and Surma, 1980; Kim et al., 2012; Price and Spiro, 1977; Schleicher and Wieland, 1986). In fact, analogous models to the one presented here have been used to analyze the rates of HIV synthesis and turnover after the administration of anti-viral drugs (Ho et al., 1995; Perelson et al., 1996) instead of de novo production. This could be possible with the presented framework but was not thoroughly tested.

## Troubleshooting

### Problem 1

Fitting quality for the mRNA is poor (i.e., the fitting function does not follow the expected shape of the curve) (step 1 of Data acquisition and step 2 of Data fitting).

### Potential solutions

Test smoothing the raw data with a small-windowed (3–5 data point) moving average. This should help when time points are sparse or experimental measures are noisy. Such small smoothing should not greatly change the downstream calculated rates (e.g., anterograde ColIV D_p_ median changes from 0.12 to 0.11 when using non-smoothed data) but could improve modeling performance as offering a better representation of the experimental mRNA dynamics.

Test a different fitting function. For example, if the mRNA profile shows a recognizable shape (e.g., Gaussian), such function could be used instead of a generic spline to achieve better fitting.

Consider increasing the temporal resolution of the acquisition. This will provide the fitting functions with more data points as input, helping to capture the observed dynamics.

### Problem 2

Multiple mRNA time-course experiments were carried out, but the code expects a single mRNA input (step 1 of Data acquisition).

### Potential solution

Average the experiments to obtain a single input

### Problem 3

The effects of photobleaching are not negligible when acquiring protein expression over time by means of fluorescence imaging (step 2 of Data acquisition).

### Potential solutions

Please do not try bleach corrections as they might skew the data. As the fluorescence is used as proxy for the protein amount, this might affect the results.

Test down-sampling by imaging with a lower temporal resolution. The signal should still show a discernable logistic behavior for the subsequent steps to work.

Consider using a different experimental acquisition mode (e.g., western blot).

### Problem 4

The confidence intervals for the synthesis and degradation rates are larger than the biological variability between replicates – this is suggesting that the modeling is failing (step 4 of Anterograde modeling and step 5 of Retrograde modeling).

### Potential solution

Check that the model assumptions have been verified, and the protein and mRNA expression observed as close as possible from initiation to homeostasis.

## Resource availability

### Lead contact

Further information and requests for resources and reagents should be directed to and will be fulfilled by the lead contact, Brian Stramer (brian.m.stramer@kcl.ac.uk).

### Materials availability

*Drosophila* strains and other reagents used in this study were generated in (Matsubayashi et al., 2020) and will be available upon reasonable request.

### Data and code availability

The sample data used in this protocol is available in the attached Excel file sample_data.xlsx. The code is available to download at https://github.com/stemarcotti/protein_turnover_modelling.

## Supplemental information

Material S1. Excel workbook to facilitate the conversion between the GraphPad parameters and the logistic parameters, related to step 3 of Data fitting

Material S2. Excel workbook to calculate protein half-life from turnover rates, related to step 6 of Calculate half-life

Material S3. Sample experimental input data for modeling, related to step 1 and step 2 of Data acquisition
